# MPC2 Overexpression Drives Mitochondrial Oxidative Phosphorylation and Promotes Progression in Diffuse Large B-Cell Lymphoma

**DOI:** 10.1007/s10528-025-11100-8

**Published:** 2025-04-27

**Authors:** Haoneng Wu, Qiuran Zhao, Xiaobo Ma, Ying Zhao, Qing Wang, Jinguang Bai, Songling Huang

**Affiliations:** 1 Yunnan Key Laboratory of Laboratory Medicine, Kunming, Yunnan China; 2 Yunnan Province Clinical Research Center for Laboratory Medicine, Kunming, Yunnan China; 3 Department of Clinical Laboratory, The First Affiliated Hospital of Kunming Medical University, No. 295 Xichang Road, Wuhua District, Kunming, 650032 Yunnan China; 4 Department of Clinical Laboratory, The Second Affiliated Hospital of Kunming Medical University, Kunming, China; 5 Department of Clinical Laboratory, The First People’s Hospital of Lushui City, Lushui City, Yunnan China

**Keywords:** Diffuse large B-cell lymphoma (DLBCL), Mitochondrial pyruvate carrier 2 (MPC2), Oxidative phosphorylation (OXPHOS), Tumor metabolism, Transcriptomics

## Abstract

Diffuse Large B-Cell Lymphoma (DLBCL) is an aggressive form of non-Hodgkin lymphoma with heterogeneous molecular characteristics. Altered metabolism, particularly mitochondrial function, has emerged as a critical factor in cancer progression. However, the role of mitochondrial metabolism in DLBCL remains poorly understood. This study aimed to identify key mitochondrial factors associated with DLBCL progression. We analyzed transcriptomic data from multiple DLBCL datasets (GSE83632, TCGA–GTEX, GSE181063, GSE4475) using differential expression analysis, weighted gene co-expression network analysis (WGCNA), and Gene Set Enrichment Analysis (GSEA). The expression and function of the identified key factor, Mitochondrial Pyruvate Carrier 2 (MPC2), were validated using clinical samples, DLBCL cell lines, and an in vivo mouse model of xenograft. Integrative bioinformatics analysis identified MPC2 as a significantly upregulated gene in DLBCL, associated with enrichment of oxidative phosphorylation (OXPHOS) and cell cycle-related genes. MPC2 overexpression was confirmed in clinical DLBCL samples and cell lines at both mRNA and protein levels. Knockdown of MPC2 in DLBCL cells impaired mitochondrial OXPHOS, increased glycolysis, and suppressed cell proliferation, invasion, and 3D spheroid formation. In vivo, MPC2 silencing significantly reduced tumor growth in a xenograft mouse model. Our findings reveal MPC2 as a key regulator of mitochondrial function in DLBCL, promoting tumor progression through enhanced OXPHOS. This study provides new insights into the metabolic reprogramming of DLBCL and suggests MPC2 as a potential therapeutic target for this aggressive lymphoma.

**Graphical Abstract:**

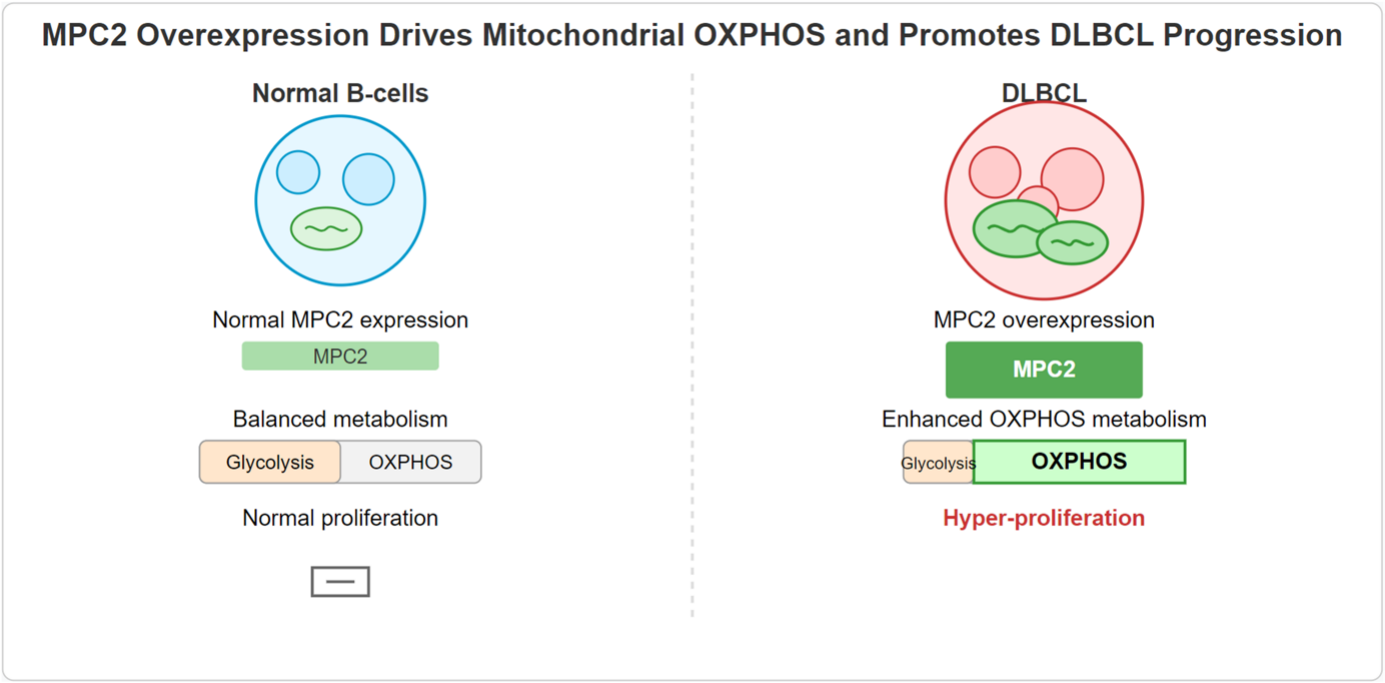

**Supplementary Information:**

The online version contains supplementary material available at 10.1007/s10528-025-11100-8.

## Introduction

Diffuse Large B-Cell Lymphoma (DLBCL) is the most common subtype of non-Hodgkin lymphoma, accounting for approximately 30–40% of all cases (Li et al. 2018). This aggressive malignancy originates from mature B lymphocytes and is characterized by the rapid proliferation of large neoplastic B cells (Pasqualucci and Dalla-Favera 2018). Diagnosis typically involves a combination of clinical presentation, histopathological examination, immunophenotyping, and molecular studies (Sehn and Salles 2021). Treatment options have evolved significantly in recent years, with the standard of care being rituximab plus cyclophosphamide, doxorubicin, vincristine, and prednisone, R-CHOP (Tilly et al. 2022). While the overall prognosis has improved with current therapies, approximately 30–40% of patients still experience relapse or refractory disease (Crump et al. 2017). Current limitations include the need for more effective treatments for high-risk and relapsed/refractory patients, as well as the development of predictive biomarkers to guide personalized therapy (Chapuy et al. 2018; Daneels et al. 2022; Goh and Ngai 2023).

Metabolic reprogramming is a hallmark of cancer, characterized by alterations in cellular metabolism to support rapid proliferation and survival (Pavlova and Thompson 2016). This phenomenon encompasses changes in various pathways, including glycolysis, glutaminolysis, and lipid metabolism (Faubert et al. xxxx). Mitochondria play a crucial role in cancer metabolism, contrary to initial beliefs that cancer cells relied solely on glycolysis (Vyas et al. 2016). Key mitochondrial alterations in cancer include TCA cycle remodeling, electron transport chain modifications, and changes in mitochondrial biogenesis (Weinberg and Chandel 2015). In DLBCL, specific mitochondrial metabolic changes have been observed, such as increased reliance on oxidative phosphorylation in certain subtypes and the importance of mitochondrial translation for cell survival (Norberg et al. 2017). BCL2 overexpression, common in DLBCL, influences both apoptosis regulation and mitochondrial metabolism (Kelly and Strasser 2011; Klanova and Klener 2020). The metabolic heterogeneity observed in DLBCL subtypes affects treatment responses and presents opportunities for targeted therapies (Caro et al. 2012). Understanding these metabolic alterations, particularly in mitochondrial function, provides new avenues for therapeutic intervention in DLBCL and other cancers.

Pyruvate, a key metabolic intermediate, plays a crucial role in mitochondrial metabolism and is involved in several important pathways (Gray et al. 2014). In the mitochondria, pyruvate can be oxidized to acetyl-CoA by pyruvate dehydrogenase (PDH), fueling the TCA cycle, and oxidative phosphorylation (Bose et al. 2019). Alternatively, pyruvate can be carboxylated to oxaloacetate by pyruvate carboxylase (PC), supporting anaplerosis and gluconeogenesis (Cheng et al. 2011). In cancer, including DLBCL, pyruvate metabolism is often dysregulated, contributing to metabolic reprogramming (Olson et al. 2016). This enhanced mitochondrial metabolism supports energy production and provides biosynthetic precursors for tumor growth. Moreover, the balance between pyruvate oxidation and carboxylation can influence tumor aggressiveness and treatment response in DLBCL (Jiang et al. 2016). Mitochondrial pyruvate carrier 2 (MPC2) functions as a mitochondrial pyruvate carrier to connect glycolysis and citrate cycle (Kuerbanjiang et al. 2021; Schell et al. 2014; Li et al. 2016; Buchanan and Taylor 2020; Nagampalli et al. 2018). In solid tumors, such as colorectal and prostate cancers, MPC2 seems to function as a tumor suppressor by inhibiting the Warburg effect and cancer cell proliferation, with their decreased expression associated with enhanced tumor growth through mechanisms involving mTOR pathway activation (Kuerbanjiang et al. 2021; Schell et al. 2014; Li et al. 2016). However, in hematological malignancies, such as DLBCL, where cells have been shown to exhibit increased pyruvate oxidation and mitochondrial respiration (Norberg et al. 2017), potentially due to unrestricted oxygen supply in the blood, the role of MPC2 in regulating their metabolism and disease progression remains to be elucidated.

In this study, we aimed to identify key mitochondrial factors associated with DLBCL progression and investigate their functional roles. Through integrative bioinformatics analysis of multiple DLBCL transcriptomic datasets, we identified MPC2 as a significantly upregulated gene correlated with OXPHOS in DLBCL. While the tumor-suppressive role of MPC2 has been established in solid tumors (Kuerbanjiang et al. 2021; Schell et al. 2014; Li et al. 2016), its function in hematological malignancies, particularly in DLBCL where mitochondrial metabolism is distinctly upregulated, remains unexplored. We validated MPC2 overexpression in clinical DLBCL samples and cell lines, and investigated its functional effects using in vitro and in vivo models. Our findings reveal a previously unrecognized role of MPC2 as a critical regulator of mitochondrial metabolism that promotes DLBCL progression by enhancing OXPHOS, contrary to its function in solid tumors. This study not only provides new insights into the metabolic reprogramming of DLBCL but also uncovers a context-dependent function of MPC2, suggesting it as a potential therapeutic target for this aggressive lymphoma.

## Methods

### Public Data Analysis

Transcriptomic data for Diffuse Large B-Cell Lymphoma (DLBCL) were obtained from the Gene Expression Omnibus (GEO) database (GSE83632, GSE181063, GSE4475) and The Cancer Genome Atlas (TCGA) combined with Genotype-Tissue Expression (GTEX) databases. Differential gene expression analysis was performed using the limma package (v3.46.0) in R (v4.1.0). Weighted gene co-expression network analysis (WGCNA) was conducted using the WGCNA package (v1.70-3) in R. Gene Set Enrichment Analysis (GSEA) was performed using the clusterProfiler package (v4.0.5) in R.

### Clinical Sample Collection

Peripheral blood samples were collected from 10 DLBCL patients and 10 healthy controls at the First Affiliated Hospital of Kunming Medical University. The study was approved by the Ethics Committee of the First Affiliated Hospital of Kunming Medical University [Approval Number (2024) Lunshen L No. 227], and all participants provided written-informed consent. CD19+ B cells were isolated using the CD19 MicroBeads Kit (Miltenyi Biotec, Bergisch Gladbach, Germany, catalog #130-050-301) according to the manufacturer's protocol. Briefly, peripheral blood mononuclear cells (PBMCs) were isolated by Ficoll–Paque density-gradient centrifugation, followed by magnetic labeling with CD19 MicroBeads. The labeled cells were then separated using a MACS Column placed in a MACS Separator.

### Cell Culture

DLBCL cell lines SU-DHL-4 and U-2932 were obtained from Procell Life Science and Technology Co., Ltd. (Wuhan, China). HBL-1 cell line was purchased from Shanghai Ruibo Biotechnology Co., Ltd. (Shanghai, China). Cell line authentication was performed using short-tandem repeat (STR) DNA profiling, and all cell lines were confirmed to be mycoplasma-negative using the MycoAlert™ Mycoplasma Detection Kit (Lonza). Cells between passages 5–10 were used for all experiments. All cells were cultured in RPMI-1640 medium (Gibco, Thermo Fisher Scientific, Waltham, MA, USA, catalog #11875093) supplemented with 10% fetal bovine serum (FBS, Gibco, catalog #10270106) and 1% penicillin–streptomycin (Gibco, catalog #15140122) at 37 °C in a humidified incubator with 5% CO_2_.

### Lentivirus-Mediated MPC2 Knockdown

Short-hairpin RNA (shRNA) targeting MPC2 and a non-targeting control shRNA were cloned into the pLenti-H1-shRNA-(GFP-Puro) vector (OBiO Technology, Shanghai, China) (Fig. S1). Lentiviral particles were produced using the MISSION® Lentiviral Packaging Mix (Sigma-Aldrich, St. Louis, MO, USA, catalog #SHP001) in HEK293T cells according to the manufacturer's instructions. For viral transduction, 2 × 10^5^ SU-DHL-4 cells were seeded in 6-well plates and infected with lentiviral particles at a multiplicity of infection (MOI) of 10 in the presence of 8 μg/mL polybrene (Sigma-Aldrich, catalog #H9268). After 48 h, the medium was replaced with fresh medium containing 2 μg/mL puromycin (Gibco, catalog #A1113803) for selection. Stable cell lines were established after two weeks of puromycin selection.

### Quantitative Real-Time PCR (qRT-PCR)

Total RNA was extracted using the RNeasy Mini Kit (Qiagen, Hilden, Germany, catalog #74104) following the manufacturer's protocol. cDNA was synthesized using the PrimeScript RT Reagent Kit (Takara Bio, Kusatsu, Japan, catalog #RR037A). qRT-PCR was performed using the SYBR Premix Ex Taq II Kit (Takara Bio, catalog #RR820A) on a LightCycler 480 II Real-Time PCR System (Roche, Basel, Switzerland). GAPDH was used as an internal control. Primer sequences were as follows: MPC2 forward 5′-TACCACCGGCTCCTCGATAA-3′, reverse 5′-ACAGCAGATTGAGCTGTGCT-3′; GAPDH forward 5′-GGAGCGAGATCCCTCCAAAAT-3′, reverse 5′-GGCTGTTGTCATACTTCTCATGG-3′. The amplification efficiency of the primers was summarized in Fig. S2.

### Immunoblotting

Cells or tumor tissues were lysed in RIPA buffer (Thermo Fisher Scientific, catalog #89900) supplemented with protease inhibitor cocktail (Roche, catalog #04693159001). Protein concentrations were determined using the BCA Protein Assay Kit (Thermo Fisher Scientific, catalog #23225). Equal amounts of protein (30 μg) were separated by 10% SDS-PAGE and transferred to PVDF membranes (Millipore, Burlington, MA, USA, catalog #IPVH00010). Membranes were blocked with 5% non-fat milk and incubated with primary antibodies overnight at 4 °C. The following primary antibodies were used: anti-MPC2 (1 μg/mL, Abcam, Cambridge, UK, catalog #ab236584) and anti-β-actin (0.2 μg/mL, Cell Signaling Technology, Danvers, MA, USA, catalog #4970). After washing, membranes were incubated with HRP-conjugated secondary antibodies (0.2 μg/mL, Cell Signaling Technology, catalog #7074 or #7076) for 1 h at room temperature. Protein bands were visualized using the ECL Western Blotting Substrate (Thermo Fisher Scientific, catalog #32106).

### Lactate Detection

Lactate levels were measured using the Lactate Colorimetric Assay Kit (BioVision, Milpitas, CA, USA, catalog #K607) according to the manufacturer's instructions. Briefly, 1 × 10^6^ cells or 10 mg of tumor tissue were homogenized in Lactate Assay Buffer. The supernatant was collected after centrifugation and incubated with the reaction mix for 30 min at room temperature. Absorbance was measured at 570 nm using a microplate reader (BioTek, Winooski, VT, USA).

### CCK-8 Proliferation Assay

Cell proliferation was assessed using the Cell Counting Kit-8 (CCK-8; Dojindo Molecular Technologies, Kumamoto, Japan, catalog #CK04). Cells (2 × 10^3^ per well) were seeded in 96-well plates. At 24, 48, 72, and 96 h, 10 μL of CCK-8 solution was added to each well and incubated for 2 h at 37 °C. Absorbance was measured at 450 nm using a microplate reader.

### Transwell Invasion Assay

Cell invasion was evaluated using 24-well Transwell chambers with 8 μm pore size (Corning, Corning, NY, USA, catalog #3422) coated with Matrigel (BD Biosciences, San Jose, CA, USA, catalog #356234). Cells (2 × 10^4^) in serum-free medium were seeded in the upper chamber, and medium containing 10% FBS was added to the lower chamber. After 24 h, invaded cells were fixed with 4% paraformaldehyde and stained with 0.1% crystal violet. Five random fields were imaged and counted under a microscope.

### Tumor Spheroid Growth Assay

Cells (1 × 10^3^) were seeded in ultra-low attachment 96-well plates (Corning, catalog #7007) in RPMI-1640 medium supplemented with 2% B-27 (Gibco, catalog #17504044) and 20 ng/mL basic fibroblast growth factor (bFGF, PeproTech, Rocky Hill, NJ, USA, catalog #100-18B). Spheroid formation was monitored for 14 days, with images captured every 3 days using an inverted microscope. Spheroid size was measured using ImageJ software (NIH, Bethesda, MD, USA).

### Oxygen Consumption Rate (OCR) Measurement

OCR was measured using the Seahorse XF Cell Mito Stress Test Kit on the Seahorse XFe96 Extracellular Flux Analyzer (Agilent Technologies, Santa Clara, CA, USA, catalog #103015-100) according to the manufacturer's protocol. Briefly, 2 × 10^4^ cells were seeded per well in a 96-well XF cell culture microplate (Agilent Technologies, catalog #102416-100) and incubated overnight. On the day of the assay, culture medium was replaced with XF base medium (Agilent Technologies, catalog #103334-100) supplemented with 10 mM glucose (Sigma-Aldrich, catalog #G8270), 1 mM pyruvate (Gibco, catalog #11360070), and 2 mM glutamine (Gibco, catalog #25030081). OCR was measured under basal conditions and in response to sequential injections of 1 μM oligomycin (Agilent Technologies, catalog #103015-100), 1 μM carbonyl cyanide-4-(trifluoromethoxy)phenylhydrazone (FCCP, Agilent Technologies, catalog #103015-100), and 0.5 μM rotenone/antimycin A (Agilent Technologies, catalog #103015-100). Results were normalized to the protein content of each sample, which was determined using the BCA Protein Assay Kit (Thermo Fisher Scientific, catalog #23225).

### In Vivo Experiment

All animal experiments were approved by the Ethical Review Committee for Animal Experiments of Kunming Medical University (Approval Number kmmu20241762), and conducted in accordance with institutional guidelines. Six-week-old female BALB/c nude mice (*n* = 5 per group) were purchased from Shanghai SLAC Laboratory Animal Co., Ltd. (Shanghai, China) and housed in a specific pathogen-free (SPF) facility with a 12-h light/dark cycle and free access to food and water. Mice were subcutaneously injected with 5 × 10^6^ SU-DHL-4 cells (control or MPC2 knockdown) in 100 μL of PBS mixed with an equal volume of Matrigel (BD Biosciences, catalog #356234) into the right flank. Tumor volume was measured every 5 days using digital calipers and calculated using the formula: volume = (length × width^2^)/2. After 35 days, mice were euthanized by cervical dislocation, and tumors were excised, weighed, and processed for further analysis.

### Statistical Analysis

All statistical analyses were performed using GraphPad Prism 8.0 (GraphPad Software, San Diego, CA, USA). Data are presented as mean ± standard deviation (SD) from at least three independent experiments. Comparisons between two groups were analyzed using two-tailed Student's *t*-test. Multiple group comparisons were performed using one-way ANOVA followed by Tukey's post hoc test. *p*-values < 0.05 were considered statistically significant.

## Results

### Profiling of Transcriptomic Changes in Diffuse Large B-Cell Lymphoma (DLBCL)

A search of the GEO database using DLBCL as the query term yielded a transcriptome microarray sequencing dataset with the ID GSE83632. This dataset comprised blood samples from 87 healthy individuals and 76 DLBCL patients. Analysis of this dataset revealed 664 downregulated genes and 685 upregulated genes.

**Fig. 1:**
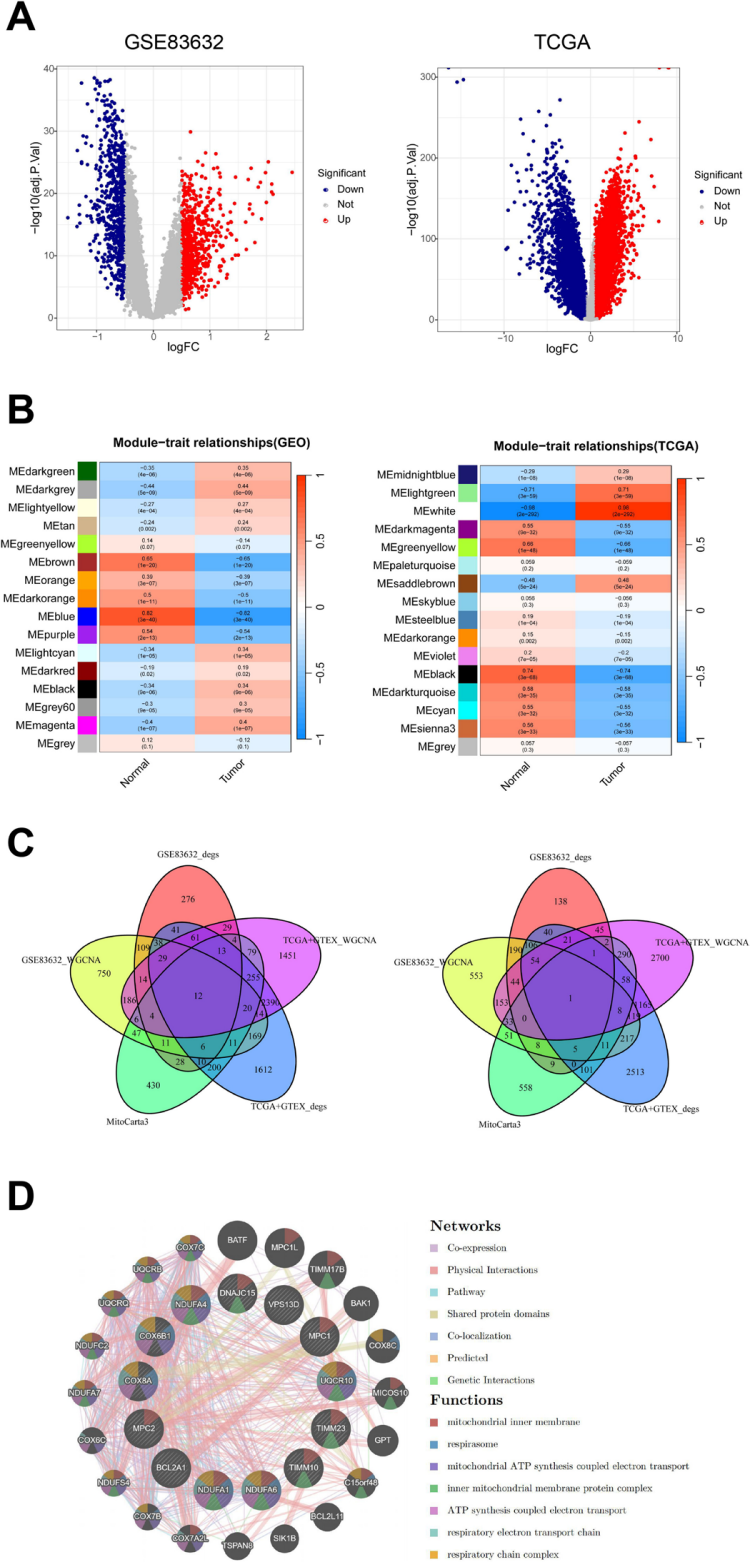
Profiling of transcriptomic changes in diffuse large B-cell lymphoma (DLBCL). **A** Volcano plots showing differentially expressed genes in DLBCL samples compared to normal samples from GSE83632 and TCGA–GTEX datasets. **B** Heatmaps of WGCNA module-trait relationships for GSE83632 and TCGA–GTEX datasets, highlighting MEblue and MEwhite modules as the most relevant gene cluster for DLBCL, respectively. **C** Venn diagram illustrating the intersection of differentially expressed genes, WGCNA module genes, and mitochondria-related genes from MitoCarta3.0. **D** Protein interaction network of intersecting genes constructed using GeneMANIA, with functional enrichment analysis results highlighted

In addition, data from the TCGA and GTEX databases, encompassing 337 normal samples and 76 DLBCL samples, were analyzed. This analysis identified 4420 downregulated genes and 5008 upregulated genes (Fig. 1A). In order to identify gene clusters associated with DLBCL, we performed WGCNA to identify DLBCL-related gene module. Analysis of the GSE83632 dataset revealed 16 distinct gene modules. Among these, the MEblue module exhibited the strongest correlation with disease phenotypes, demonstrating an absolute correlation coefficient of 0.82 (|cor|= 0.82) and a *p* value of 3e−40. This module encompasses 1553 genes. Similarly, analysis of the combined GTEX and TCGA datasets also yielded 16 gene modules. In this dataset, the MEwhite module showed the most significant association with disease traits, with an absolute correlation coefficient of 0.98 (|cor|= 0.98) and an extremely low *p*-value of 2e−292. This module comprises 4694 genes (Fig. 1B). Furthermore, mitochondria-related genes were downloaded from the MitoCarta3.0 database and intersected with both the WGCNA module genes and differentially expressed genes. The intersection analysis identified 12 upregulated genes and 1 downregulated gene common to all three sets (Fig. 1C). A protein interaction network was constructed for the intersecting genes using the GeneMANIA database. Functional enrichment analysis was performed on this interaction network, revealing alterations in functional genes associated with the mitochondrial membrane and electron transport chain (Fig. 1D).

### Identification of MPC2 as a Key Mitochondrial Functional Factor Associated with DLBCL

**Fig. 2:**
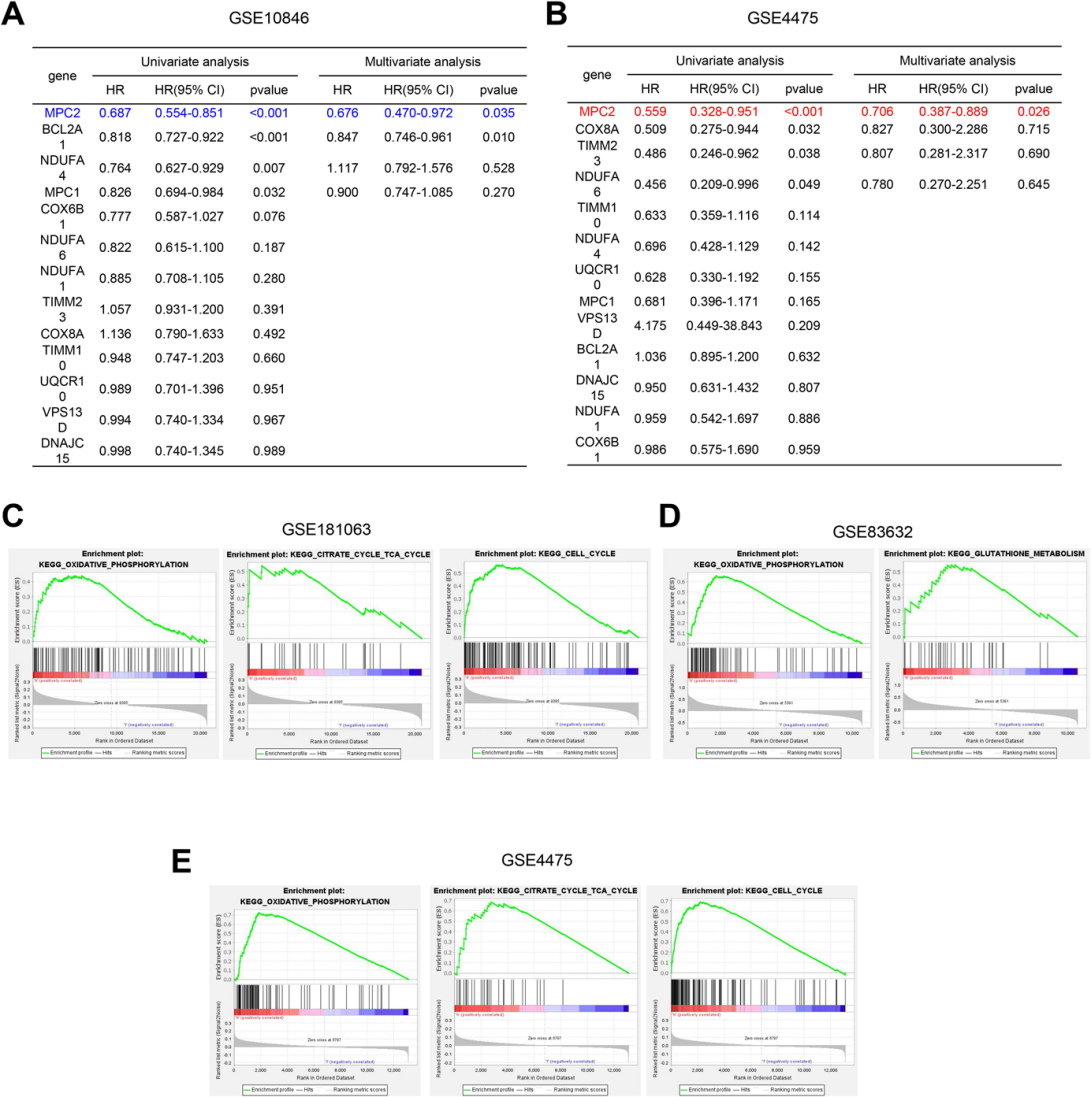
Identification of MPC2 as a key mitochondrial functional factor associated with DLBCL. **A** and **B** Univariate and multivariate Cox regression analysis for DLBCL-associated mitochondrial genes in GSE83632 and TCGA–GTEX datasets. **C**–**E** GSEA plots showing enrichment of mitochondrial function-related and cell cycle-related gene sets in MPC2 high-expression groups from GSE181063, GSE83632, and GSE4475 datasets. Red color denotes genes positively related to MPC2 expression, and blue color represents genes negatively related to MPC2 expression. The orange line represents cumulative enrichment scores (Color figure online)

Next, both univariate and multivariate Cox regression analyses were performed to identity risk factor associated with DLBCL. In both datasets, MPC2 was significantly associated with DLBCL (Fig. 2A, B). To show the relevance of MPC2 expression of mitochondrial functions, transcriptomic data for DLBCL from GSE181063, GSE83632, and GSE4475 datasets were analyzed. Samples in each dataset were divided into MPC2 high-expression and low-expression groups based on the median expression level of MPC2. Gene Set Enrichment Analysis (GSEA) revealed that the MPC2 high-expression group showed enrichment in mitochondrial function-related genes, including those involved in oxidative phosphorylation (OXPHOS) and the citrate cycle, as well as cell cycle-related genes (Fig. 2C–E). These data suggest MPC2 as a key mitochondrial functional factor associated with DLBCL progression.

### Validation of MPC2 Expression in Clinical Samples

**Fig. 3:**
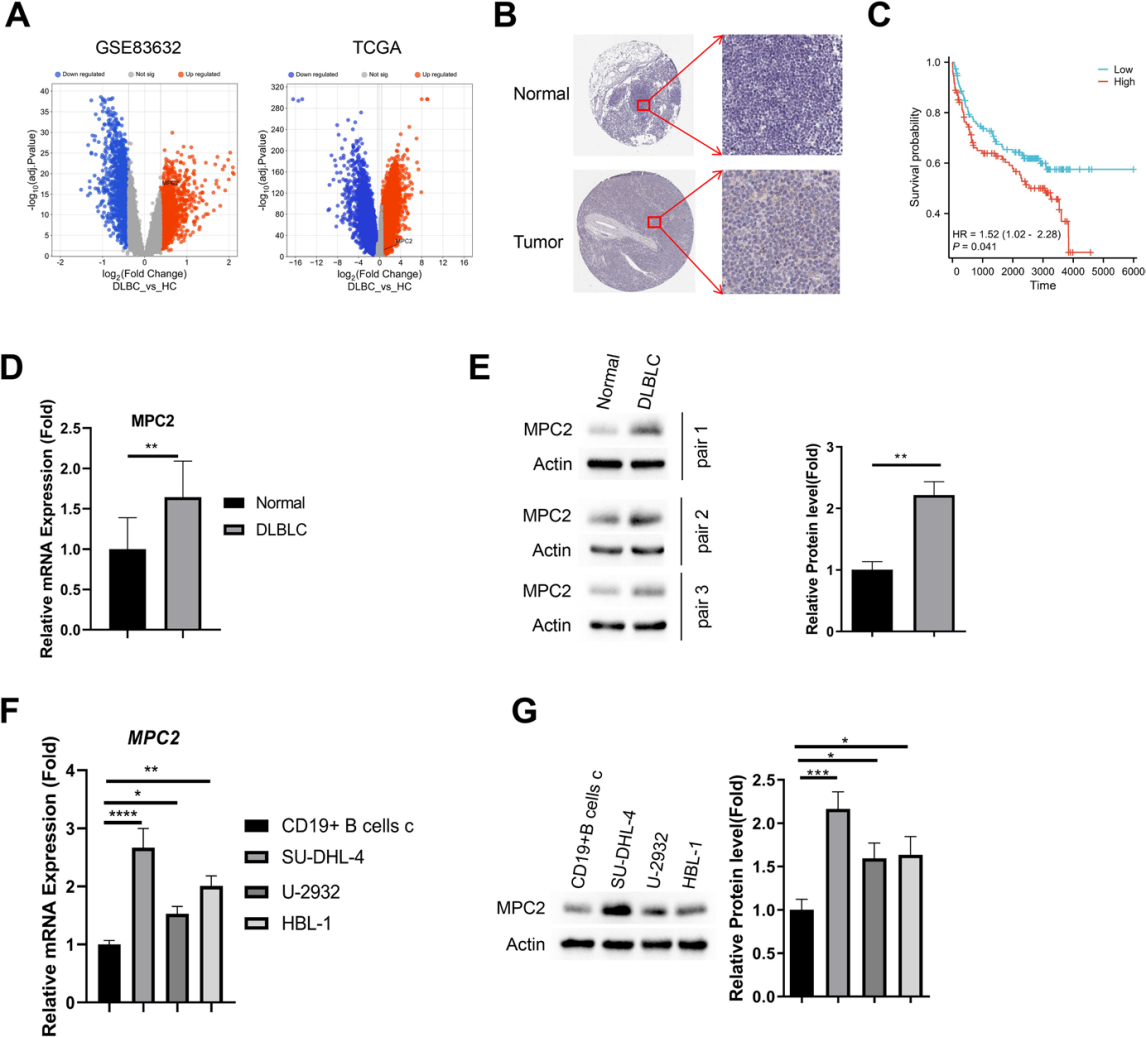
Validation of MPC2 expression in clinical samples. **A** Box plots comparing MPC2 expression levels between normal and DLBCL samples in GSE83632 and GTEX–TCGA datasets. **B** Immunohistochemical staining images of MPC2 in normal and DLBCL bone marrow samples from the Human Protein Atlas database. **C** Survival analysis of DLBCL patients from GTEX–TCGA dataset based on the expression levels of MPC2. **D** qRT-PCR results showing MPC2 mRNA levels in peripheral blood samples from normal controls and DLBCL patients (*n* = 10 samples in each category). **E** Western blot analysis of MPC2 protein levels in peripheral blood samples from normal controls and DLBCL patients. **F** qRT-PCR results comparing MPC2 mRNA levels between normal CD19+ B cells and DLBCL cell lines (SU-DHL-4, U-2932, HBL-1). **G** Western blot analysis comparing MPC2 protein levels between normal CD19+ B cells and DLBCL cell lines. *n* = 3 experiments. *p < 0.05; **p < 0.01; ***p < 0.001; ****p < 0.0001

MPC2 expression was found to be significantly upregulated in DLBCL samples compared to normal samples, in both GSE83632 and GTEX–TCGA dataset (Fig. 3A). Immunohistochemical staining data from Human Protein Atlas (HPA) database also showed the elevation of MPC2 protein level in bone marrow samples of DLBCL patients (Fig. 3B). Furthermore, DLBCL patients with high levels of MPC2 expression showed a reduced overall survival (Fig. 3C). We further collected peripheral blood samples from normal controls and DLBCL patients. Both qRT-PCR analysis and western blot results confirmed the upregulation of MPC2 in DLBCL samples (Fig. 3D, E). Moreover, compared to CD19+ B cells isolated from normal blood samples, MPC2 expression was also significantly increased at mRNA and protein levels in multiple DLBCL cell lines, including SU-DHL-4, U-2932 and HBL-1 (Fig. 3F, G).

### Knocking Down MPC2 Impairs Mitochondrial OXPHOS and Suppresses the Malignancy of DLBCL Cells

**Fig. 4:**
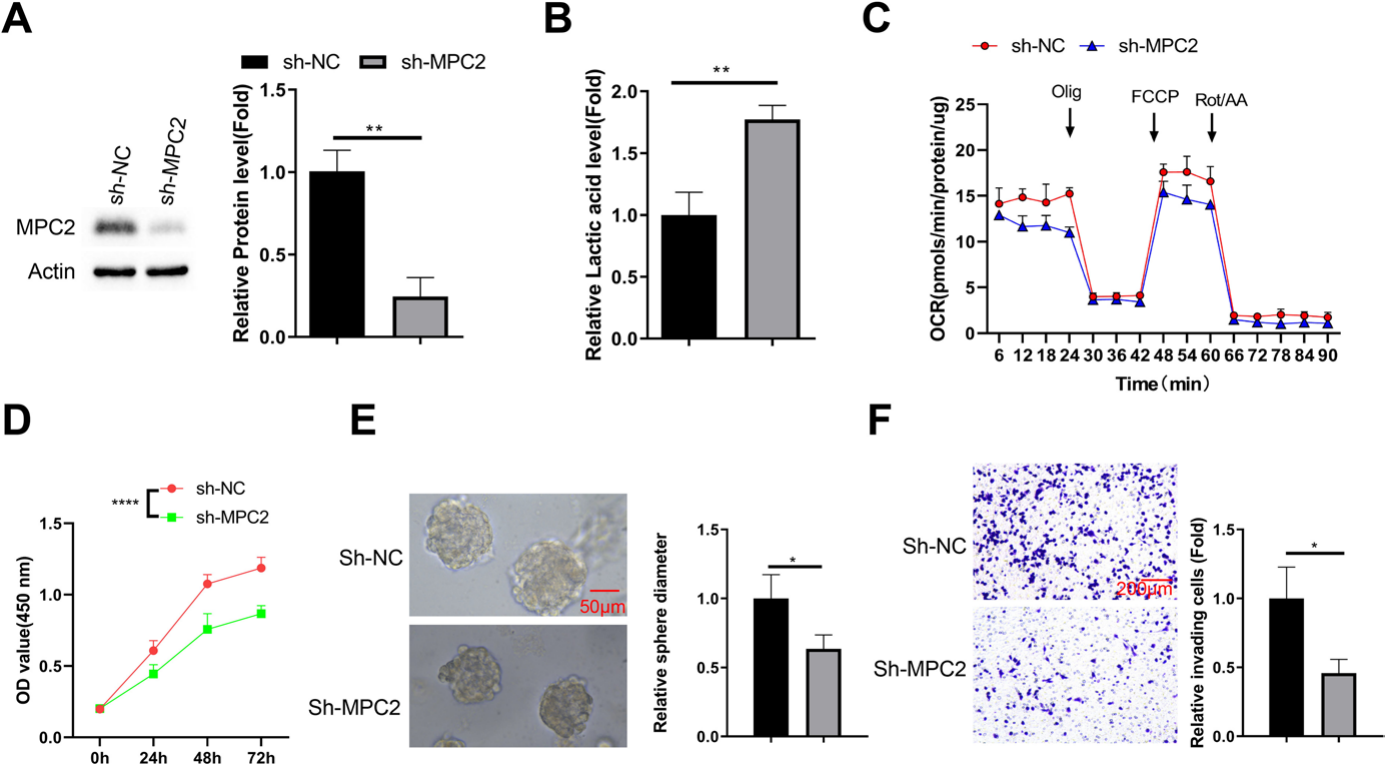
Knocking down MPC2 impairs mitochondrial OXPHOS and suppresses the malignancy of DLBCL cells. **A** Western blot confirming MPC2 knockdown in SU-DHL-4 cells transduced with sh-MPC2 lentivirus. **B** Bar graph showing lactate production levels in control and MPC2 knockdown SU-DHL-4 cells. **C** Line graph depicting oxygen consumption rate (OCR) in control and MPC2 knockdown SU-DHL-4 cells. **D** Growth curves of control and MPC2 knockdown SU-DHL-4 cells as measured by CCK-8 assay. **E** Representative images and quantification of 3D tumor spheroid formation by control and MPC2 knockdown SU-DHL-4 cells. **F** Representative images and quantification of Transwell invasion assay for control and MPC2 knockdown SU-DHL-4 cells. *n* = 3 experiments. *p < 0.05; **p < 0.01; ***p < 0.001; ****p < 0.0001

To demonstrate the essential role of MPC2 overexpression in DLBCL cells, we applied lentivirus carrying sh-NC (control shRNA) or sh-MPC2 (shRNA targeting MPC2) to transduce SU-DHL-4 cells. Transduction with lentivirus carrying sh-MPC2 resulted in stable knockdown of PMC2 in DLBCL cells (Fig. 4A). Since MPC2 functions as a mitochondrial pyruvate carrier to connect glycolysis and citrate cycle (Buchanan and Taylor 2020; Nagampalli et al. 2018; Ruiz-Iglesias and Mañes 2021), we analyzed the rate of glycolysis by detecting lactate production level and mitochondrial OXPHOS activity by detecting oxygen consumption rate (OCR). Upon MPC2 knockdown, there was an increase in lactate level in DLBCL cells, indicating the preferential conversion of pyruvate into lactate (Fig. 4B). Concurrently, MPC2 knockdown led to a reduction of OCR in mitochondria (Fig. 4C). CCK-8 assay further showed that MPC2 silencing suppressed cell proliferation of SU-DHL-4 cells (Fig. 4D). In the 3D period growth assay, silencing of MPC2 reduced the size of tumor spheroid formation (Fig. 4E). Meanwhile, MPC2 knockdown attenuated the invasive capacity of SU-DHL-4 cells, as revealed by an decrease of invading cells in Transwell invasion assay (Fig. 4F). Together, these data indicate that a high level of MPC2 expression is required for maintaining mitochondrial OXPHOS activity and malignant features in DLBCL cells.

### Knocking Down MPC2 Impedes Tumorigenesis of DLBCL Cells in Nude Mice

**Fig. 5:**
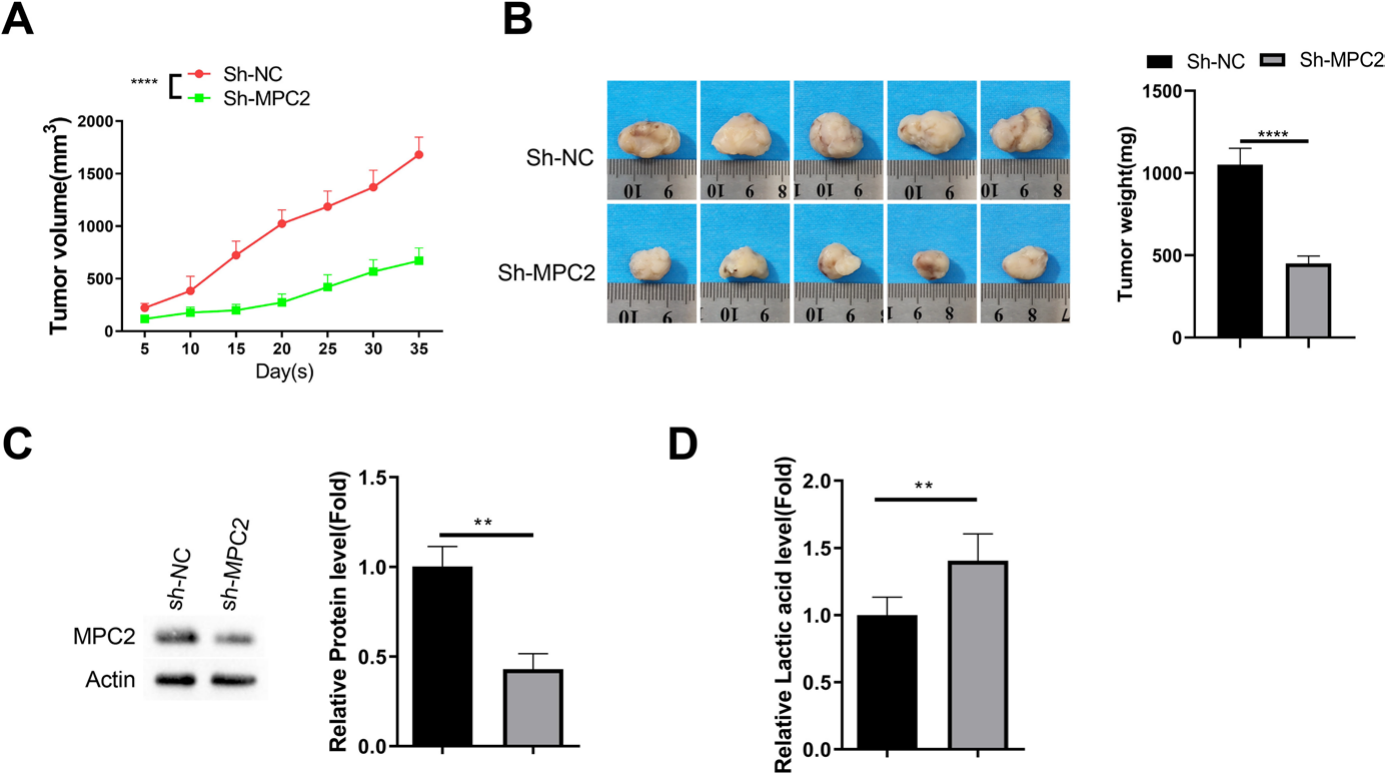
Knocking down MPC2 impedes tumorigenesis of DLBCL cells in nude mice. **A** Tumor growth curves of nude mice inoculated with control (sh-NC) or MPC2 knockdown (sh-MPC2) SU-DHL-4 cells. **B** Bar graph comparing tumor weights between control and MPC2 knockdown groups at the end of the experiment. **C** Western blot confirming MPC2 knockdown in tumor tissues derived from sh-MPC2 SU-DHL-4 cells. **D** Bar graph showing lactate levels in tumor tissues from control and MPC2 knockdown groups. *n* = 5 animal in each group. *p < 0.05; **p < 0.01; ***p < 0.001; ****p < 0.0001

Inspired by the in vitro findings, we further inoculated sh-NC and sh-MPC2 SU-DHL-4 cells into nude mice. Tumor growth curves showed an impaired tumor formation by SU-DHL-4 cells with MPC2 knockdown (Fig. 5A). Furthermore, the tumor weights were reduced in sh-MPC2 group (Fig. 5B). The silencing of MPC2 was confirmed by western blot in tumor tissues from SU-DHL-4 cells with MPC2 knockdown (Fig. 5C). We also measured the lactate levels in the tumor tissues, which demonstrated the over-accumulation of lactate after MPC2 knockdown (Fig. 5D). Therefore, these data suggest that targeting MPC2 can impede tumor formation of DLBCL cells.

## Discussion

In this study, we identified MPC2 as a key regulator of mitochondrial metabolism in DLBCL. Our integrative bioinformatics analysis revealed MPC2 as a significantly upregulated gene in DLBCL, associated with enrichment of OXPHOS and cell cycle-related genes. Functionally, knockdown of MPC2 in DLBCL cells impaired mitochondrial OXPHOS, increased glycolysis, and suppressed cell proliferation, invasion, and 3D spheroid formation. Importantly, MPC2 silencing significantly reduced tumor growth in a xenograft mouse model. These findings highlight the critical role of MPC2 in promoting DLBCL progression through enhanced OXPHOS and suggest that targeting MPC2 may represent a novel therapeutic strategy for DLBCL. Our study provides new insights into the metabolic reprogramming of DLBCL and underscores the importance of mitochondrial metabolism in lymphoma biology.

The role of mitochondrial pyruvate carrier (MPC) in cancer metabolism has garnered significant attention in recent years, with our findings on MPC2 in DLBCL contributing to this growing body of knowledge. MPC plays a crucial role in regulating pyruvate entry into mitochondria, thereby influencing the balance between glycolysis and oxidative phosphorylation (Ruiz-Iglesias and Mañes 2021). Our observation that MPC2 knockdown impairs OXPHOS and increases glycolysis in DLBCL cells aligns with previous studies in other cancer types. For instance, Li et al. reported that reduced MPC function is associated with a Warburg phenotype in esophageal squamous cell carcinomas, characterized by increased lactate production (Li et al. 2017). This is consistent with our findings in DLBCL, where MPC2 silencing led to increased lactate levels. Moreover, the importance of MPC in cancer progression has been demonstrated across various malignancies. Bensard et al. showed that MPC regulates tumor initiation in multiple cancer types, highlighting its potential as a therapeutic target (Bensard et al. 2020). In the context of lymphomas, Wei et al. recently demonstrated that mitochondrial pyruvate supports lymphoma proliferation by fueling a glutamate pyruvate transaminase 2-dependent glutaminolysis pathway (Wei et al. xxxx), further emphasizing the critical role of pyruvate metabolism in lymphoid malignancies.

Our findings on the pro-tumorigenic role of MPC2 in DLBCL may seem paradoxical given some reports suggesting tumor-suppressive functions of MPC in other cancers (Xue et al. 2021). However, this apparent discrepancy underscores the context-dependent nature of metabolic reprogramming in cancer. The specific metabolic requirements of DLBCL cells may explain why enhanced MPC2 expression and OXPHOS contribute to tumor progression in this particular malignancy. This is supported by Jiang et al., who demonstrated that metabolic reprogramming involving pyruvate metabolism plays a crucial role in rituximab resistance in DLBCL (Jiang et al. 2021). Furthermore, the potential of targeting MPC in cancer therapy has been explored in a previous study. The authors showed that interrupting lactate uptake by inhibiting mitochondrial pyruvate transport has direct antitumor and radiosensitizing effects (Corbet et al. 2018). Interestingly, studies by Ohashi et al. and Takaoka et al. have shown that MPC modulates epithelial–mesenchymal transition in cholangiocarcinoma and controls cancer radioresistance, respectively (Ohashi et al. 2018; Takaoka et al. 2019). These findings, together with our results, suggest that modulating MPC2 activity could be a promising therapeutic strategy in DLBCL, potentially enhancing the efficacy of existing treatments or overcoming drug resistance.

Mitochondrial metabolism has emerged as a critical factor in cancer progression and drug resistance, particularly in hematological malignancies like DLBCL (Zhang et al. 2023). Our findings on the pro-tumorigenic role of MPC2 in DLBCL align with the growing body of evidence highlighting the importance of mitochondrial metabolic reprogramming in lymphoma biology. Mitochondrial metabolism has been recognized as a power hub in lymphoma progression, suggesting that targeting mitochondrial metabolism could be a promising therapeutic approach (Deberardinis 2012). Increasing evidence has also pinpointed a crucial role of mitochondrial bioenergetics in drug resistance in hematological malignancies (Barbato et al. 2020). Our observation that MPC2 knockdown impairs OXPHOS and reduces tumor growth is consistent with the metabolic compartmentalization in DLBCL described by Gooptu et al. (2017), in which the authors showed that both mitochondrial and glycolytic metabolic pathways are necessary for DLBCL.

Recent studies have explored various strategies to target mitochondrial metabolism in lymphomas. For instance, Sharon et al. demonstrated that inhibition of mitochondrial translation could overcome venetoclax resistance in AML (Sharon et al. xxxx), while Donati et al. showed that targeting mitochondrial respiration in combination with BCL2 inhibition is effective against high-grade MYC-associated B-cell lymphoma (Donati et al. 2022). Furthermore, Schwarzer et al. and Yao et al. have reported promising results in targeting aggressive B-cell lymphomas through modulation of mitochondrial proteins and OXPHOS inhibition, respectively (Schwarzer et al. 2023; Yao et al. 2022). Interestingly, Cai et al. recently showed that α-ketoglutarate can inhibit DLBCL tumor growth by inducing ROS and TP53-mediated ferroptosis (Cai et al. 2023), highlighting the complex interplay between mitochondrial metabolism and cell death pathways. These studies, along with our findings on MPC2, underscore the potential of targeting mitochondrial metabolism as a therapeutic strategy in DLBCL and other hematological malignancies.

While our study provides valuable insights into the role of MPC2 in DLBCL, several limitations and future directions should be considered. A key limitation is the lack of comprehensive clinical data correlating MPC2 expression with patient outcomes and drug resistance. Future research should focus on examining these associations in large patient cohorts to establish MPC2 as a potential prognostic biomarker. In addition, the mechanisms underlying MPC2 upregulation in DLBCL remain to be fully elucidated, warranting investigation into transcriptional and post-transcriptional regulators. Further exploration of MPC2's interaction with other metabolic pathways could provide a more comprehensive understanding of DLBCL metabolism. Moreover, our use of immunodeficient nude mice models, while suitable for evaluating tumor cell-intrinsic effects, precluded the investigation of MPC2's potential impact on the immune microenvironment, which would require immunocompetent mouse models. Furthermore, while our findings are based primarily on cell lines and animal models, validation in a larger set of clinical samples would strengthen their translational relevance. The impact of tumor heterogeneity on MPC2 expression and function also warrants further investigation.

## Conclusion

To conclude, our study establishes MPC2 as a critical regulator of DLBCL metabolism and tumor progression. We demonstrated MPC2 upregulation in DLBCL and showed that its knockdown impairs OXPHOS, reduces cell proliferation, and inhibits tumor growth. These findings highlight MPC2 as a potential therapeutic target in DLBCL. Future research should focus on exploring MPC2's clinical relevance, particularly its value as a prognostic biomarker through large-scale patient cohort studies, its role in drug resistance, and developing specific inhibitors. Investigating MPC2 upregulation mechanisms and its metabolic interactions will further our understanding of DLBCL metabolism, potentially leading to novel therapeutic strategies.

## Supplementary Information

Below is the link to the electronic supplementary material.

Supplementary file1 (DOCX 455 kb)

Supplementary file2 (DOCX 168 kb)

## Data Availability

The data generated in this study are available upon request to the corresponding author. All authors are aware of and have consented to publication.
