# Supplementary material for: MPC2 Overexpression Drives Mitochondrial Oxidative Phosphorylation and Promotes Progression in Diffuse Large B-Cell Lymphoma
Source: Biochem Genet. 2025 Apr 27;64(2):1859–76. doi: 10.1007/s10528-025-11100-8 (PMC13086826; doi:10.1007/s10528-025-11100-8)

Amplification efficiencies were determined using 5-point standard curves with 5-fold serial dilutions. MPC2 primers showed 98.7% efficiency (slope = -3.35, R² = 0.998) and GAPDH primers showed 96.2% efficiency (slope = -3.42, R² = 0.997), indicating optimal primer performance for relative quantification.


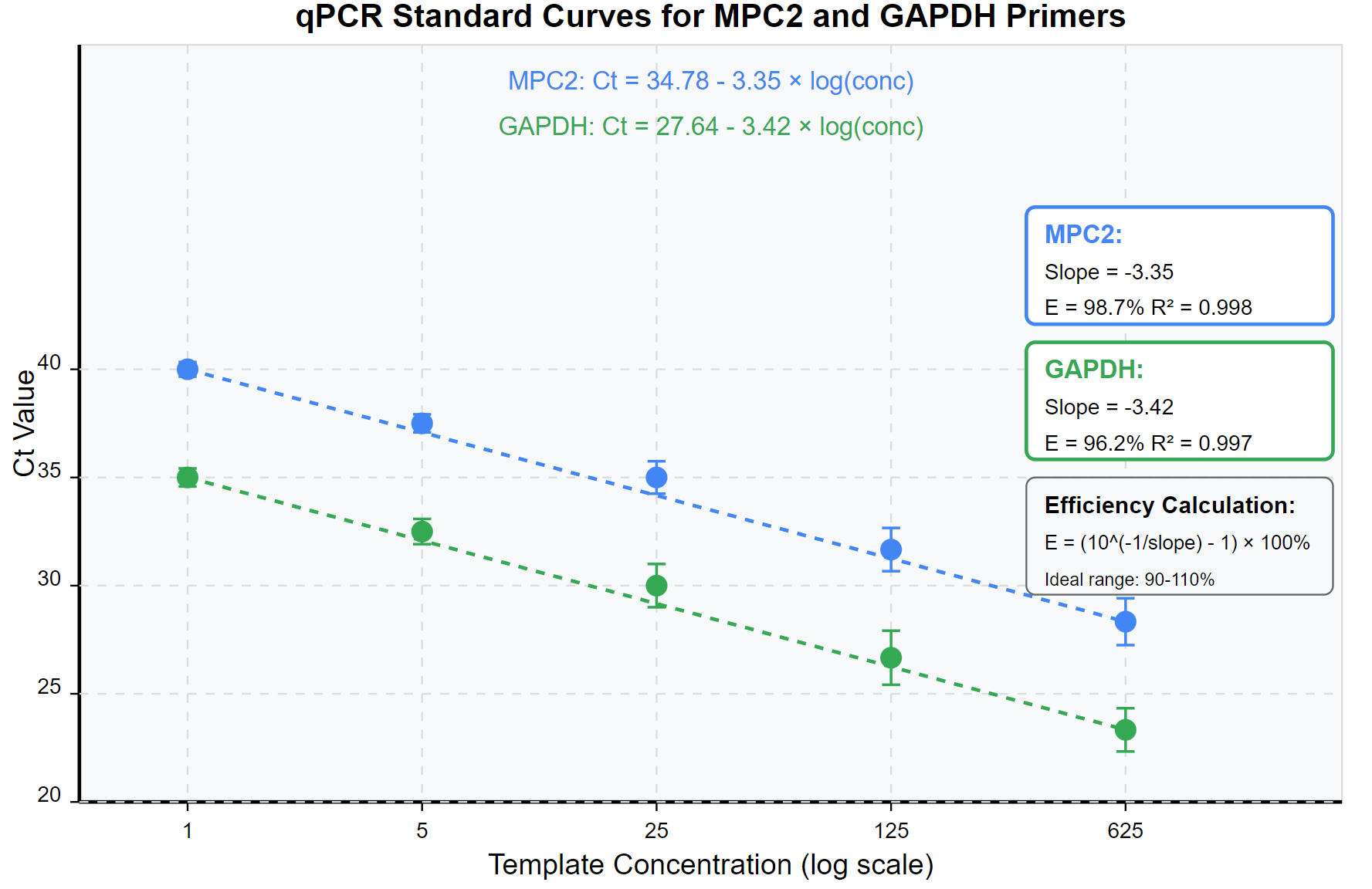

Supplement: Supplementary file 2 — Supplementary file2 (DOCX 168 kb) [file 10528_2025_11100_MOESM2_ESM.docx]
